# Screening of plant immune elicitors and their control effects on tomato yellow leaf curl virus disease

**DOI:** 10.3389/fpls.2026.1834029

**Published:** 2026-05-26

**Authors:** Dandan Wang, Sichao Liu, Yikang Xue, Shaonan Wang, Lianfen Qi, Lingdi Dong

**Affiliations:** 1 Shijiazhuang Academy of Agriculture and Forestry Sciences, Shijiazhuang, China; 2 Chengde Vegetable Technology Promotion Station, Chengde, China; 3 Institute of Cash Corps, Hebei Academy of Agriculture and Forestry Sciences, Shijiazhuang, China

**Keywords:** amino-oligosaccharin, defense enzyme, immune elicitor, oligosaccharin-chain protein, plant hormone, tomato yellow leaf curl virus

## Abstract

Tomato yellow leaf curl virus disease (TYLCV) is a devastating disease in tomato industry development. Due to the lack of efficient chemical control agents, the prevention and control of this disease has become a major challenge in the industry. This study used the self-bred tomato variety “Nongbo Fen 18109” as experimental material. Through determination of endogenous hormone contents, defense enzyme activities, and viral load in tomato leaves as core indicators, screening experiments of plant immune elicitors were conducted, and further evaluation of the selected elicitors’ field control effects on TYLCV was performed. The results showed that 5% amino-oligosaccharin aqueous solution and 6% oligosaccharin-chain protein wettable powder could significantly regulate the balance of tomato endogenous hormones, increase indole-3-acetic acid (IAA), salicylic acid (SA) contents and peroxidase (POD) activity, reduce jasmonic acid (JA) and abscisic acid (ABA) levels, and effectively enhance plant resistance to TYLCV. When used in combination, these two elicitors achieved the best control effect, with only 6 diseased plants during the experimental period and an average CT value of 14.34 in virus detection, significantly superior to single elicitor treatments and the chemical agent moroxydine hydrochloride-copper treatment. This study identified an efficient TYLCV immune elicitor combination and its application effects, providing theoretical basis and practical technical support for constructing a green prevention and control system for tomato yellow leaf curl virus disease.

## 1 Introduction

Tomato is a solanaceous fruit vegetable widely cultivated worldwide and one of the main protected horticultural crops in China. Its industry development is of great significance for ensuring vegetable market supply and increasing farmers’ economic income (Sun et al., 2016; Wang et al., 2023). In recent years, with the continuous expansion of protected tomato cultivation area, the intensification of continuous cropping obstacles, and the frequent cross-regional circulation of seedlings, the occurrence of tomato viral diseases has shown an increasing trend year by year, among which tomato yellow leaf curl virus disease is one of the most serious viral diseases (Wang et al., 2025a).TYLCV belongs to the genus Begomovirus in the family Geminiviridae, mainly transmitted by whiteflies (Bemisia tabaci) in a persistent manner. Infected plants show symptoms of leaf yellowing, curling, stunting, flower and fruit drop, and severe cases may result in whole plant death. Infected fields may suffer yield losses of 30%-80%, or even total crop failure (Moriones and Navas-Castillo, 2000). It is noteworthy that TYLCV exists as both monopartite (single DNA-A component of ~2.7-2.8 kb) and bipartite (DNA-A and DNA-B components) forms, with distinct geographic distributions. The monopartite TYLCV, prevalent in the Mediterranean region, Middle East, and Asia including China, is predominantly associated with devastating epidemics in these areas (Czosnek and Laterrot, 1997; Kheyr-Pour et al., 1991). In China, the major epidemic strain is Tomato yellow leaf curl China virus (TYLCCNV), a typical monopartite begomovirus often associated with a betasatellite (Lefeuvre et al., 2010; Moriones and Navas-Castillo, 2000).

Currently, control measures for tomato yellow leaf curl virus disease in production are relatively limited, with no specific therapeutic agents available. After disease occurrence, agricultural measures such as removing diseased plants and cleaning fields are usually adopted, or insecticides such as thiamethoxam and spirotetramat are sprayed to block whitefly transmission pathways (Liu et al., 2023). However, whiteflies reproduce rapidly and develop resistance quickly, making chemical control effects limited. Moreover, excessive use of chemical pesticides can cause pesticide residues, ecological environment damage, and pest resurgence (Nicaise, 2014), which contradicts the requirements of modern agricultural green development. Therefore, starting from improving tomato plant’s own disease resistance, utilizing plant immune induction technology to activate the plant’s innate defense system has become an important research direction for TYLCV control.

Plant immune elicitors are a class of biological or biomimetic agents that can induce systemicacquired resistance (SAR) and induced systemic resistance (ISR) in plants. They do not have directbactericidal or antiviral effects themselves, but enhance plant resistance to pests and diseases byregulating plant endogenous hormone metabolism and activating defense enzyme systems (Walters et al., 2013). These agents have advantages such as environmental friendliness, specific targets, and low risk of resistance development, and have been applied in green prevention and control of diseases in various crops (Ji et al., 2017; Wang et al., 2025a, 2016; Yin et al., 2016). Eugenol can induce the accumulation of salicylic acid and nitric oxide in tomato and up-regulate the expression of TYLCV-specific resistance gene SlPer1, with control efficacy reaching 77.4%, significantly superior to conventional chemical agent moroxydine hydrochloride (Sun et al., 2016). Novel RNA nanobiopesticides and fungal culture filtrate F8 have also been confirmed to effectively control TYLCV and other tomato viral diseases through systemic movement or induced defense responses (Liu et al., 2026), providing important foundations for developing environmentally friendly, specifically targeted, and resistance-free green control technologies. Amino-oligosaccharin and oligosaccharin-chain protein, as new biological pesticides, have been proven to effectively induce plant stress resistance in vegetables, fruit trees, and other crops (Jia et al., 2016), but their separate and combined application effects in tomato TYLCV control have not yet been clarified.

Based on this, this study used the tomato variety “Nongbo Fen 18109” as material to screen plant immune elicitors with high-efficiency induced resistance to TYLCV, and explore the control effects and mechanisms of optimal elicitor combinations, aiming to provide new technical solutions for green and efficient control of tomato yellow leaf curl virus disease and promote sustainable development of the tomato industry.

## 2 Materials and methods

### 2.1 Experimental materials

The experiment was conducted using soil cultivation with standardized irrigation and fertilization management. The experimental material was the “Nongbo Fen 18109” tomato variety self-bred by Shijiazhuang Academy of Agricultural and Forestry Sciences. Four plant immune elicitors were used: 5% amino-oligosaccharin aqueous solution (chitosan oligosaccharide), purchased from Shanghai Hulian Biological Medicine (Xiayi) Co., Ltd.; 6% oligosaccharin-chain protein wettable powder (protein-based), purchased from Hebei Zhongbaolv Agricultural Crop Technology Co., Ltd.; 5% S-ABA (abscisic acid), purchased from Huazhi Hebei Biotechnology Co., Ltd.; and Kang’erjian Plant Vaccine, purchased from Guangzhou Nongqiaoshi Fertilizer Co., Ltd.

### 2.2 Experimental design

#### 2.2.1 Screening experiment of different immune elicitors

**Figure 1:**
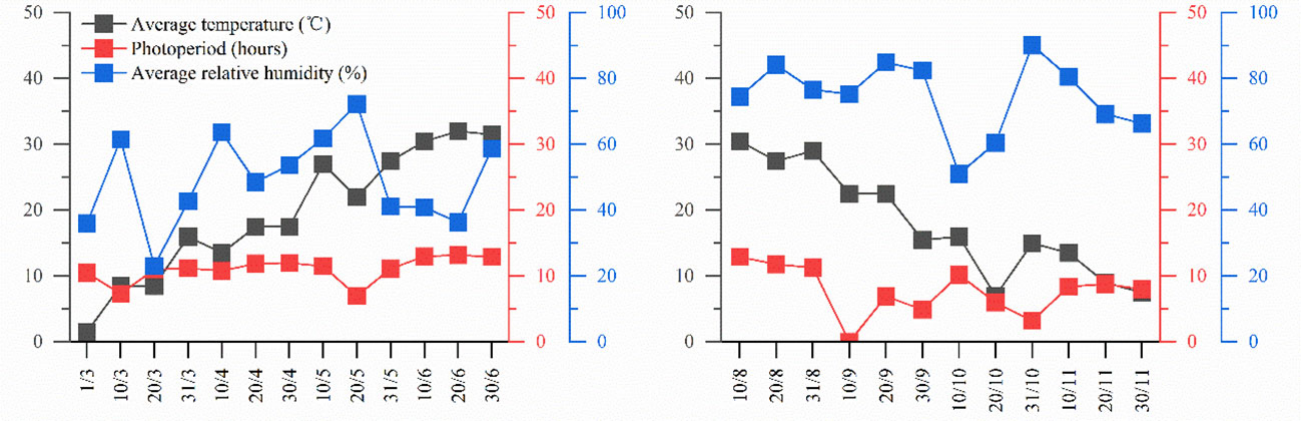
Average temperature, photoperiod, and average relative humidity in tomato growing season.

On March 1, 2024, tomato seedlings at the three-leaf and one-heart stage were transplanted into Greenhouse No. 4 at the Zhao County Base of our academy. **Figure 1** shows average temperature, photoperiod, and average relative humidity in tomato growing season. Thirty days after transplanting, tomato plants were inoculated using a previously reported infectious clone (Bai et al., 2015) (GenBank: KF612971.1). The viral isolate used in this study represents the predominant TYLCV type distributed in northern China, which contains a single genomic DNA component of approximately 2.7-2.8 kb, consistent with the typical genome size of monopartite begomoviruses.” The EHA105 strain of *Agrobacterium tumefaciens* carrying the TYLCV infectious clone was grown for 48 hours in LB medium supplemented with 100 mg·L^-^¹ rifampicin (Rif) and 50 mg·L^-^¹ kanamycin sulfate (Kan). The bacterial suspension was then centrifuged at 4 °C and 5000 rpm for 5 minutes. The resulting pellet was resuspended and diluted in an inoculation buffer composed of 10 mmol·L^-^¹ MES, 10 mmol·L^-^¹ MgCl_2_, and 150 μmol·L^-^¹ acetosyringone. According to the method described by Huang et al. (2022), the diluted bacterial suspension (adjusted to an OD600 value of 0.6–0.8) was prepared and injected into tomato leaves using a needleless syringe. For each treatment, at least ten tomato plants were inoculated, and data from three replicates were collected and calculated. After TY virus inoculation, leaves were observed daily for symptoms. After symptom appearance, immune elicitors were sprayed on plant leaves.

The experiment set up 5 treatments: A: 5% amino-oligosaccharin aqueous solution; B: 6% oligosaccharin-chain protein wettable powder; C: 5% S-ABA; D: Kang’erjian Plant Vaccine; E: Control (clean water). Each treatment was sprayed once every 3 days, for a total of 3 times, with 3 replicates. Sampling began 7 days after elicitor spraying, collecting the 3rd or 4th leaf from the top, rapidly freezing in liquid nitrogen, and storing at -80 °C for later use. Subsequent sampling was conducted every 7 days, for a total of 4 samplings. By measuring various physiological and viral indicators, elicitors with excellent TYLCV control effects were screened to provide material basis for subsequent field control experiments.

#### 2.2.2 Control effect experiment on TYLCV

On August 5, 2024, tomato seedlings at the three-leaf and one-heart stage were transplanted into the greenhouse at the South Campus of our academy. Forty days after transplanting, before virus disease occurrence, selected elicitors and control agents were sprayed on tomato plant leaves. The experiment set up 5 treatments: T1 (5% amino-oligosaccharin aqueous solution), T2 (6% oligosaccharin-chain protein wettable powder), T3 (5% amino-oligosaccharin + 6% oligosaccharin-chain protein, combined), T4 (moroxydine hydrochloride-copper, chemical control), T5 (clean water, blank control). Each treatment was planted with 20 tomato seedlings, isolated separately with 40-mesh insect-proof nets. The TYLCV grading criteria of Huang et al. (2025) were adopted to regularly investigate the incidence of TYLCV and detect the viral load in plant tissues.

Disease index = Σ (Number of diseased plants at every level × Level value)/(Total survey plants × the highest value).

Control effect = [(Disease index of the control group − Disease index of the treatment group)/Disease index of the control group] × 100%.

### 2.3 Determination indicators and methods

Endogenous hormone contents including indole-3-acetic acid (IAA), gibberellin (GA_3_), Trans-Zeatin Riboside (TZR), salicylic acid (SA), jasmonic acid (JA), and abscisic acid (ABA), as well as activities of peroxidase (POD), catalase (CAT), and superoxide dismutase (SOD) were determined using kits (Solarbio, Beijing) by Nanjing Ruiyuan Biotechnology Co., Ltd. (ProNet Biotech Co., Ltd.). Tomato yellow leaf curl virus load and field incidence were also determined. Hormone contents were determined according to the method of Zhang et al (Zhang et al., 2020), and enzyme activities according to the method of Wang et al (Wang et al., 2026).

#### 2.3.1 Hormone content determination method

All samples were ground to powder in liquid nitrogen. Accurately weighed samples were placed in test tubes, 10 mL acetonitrile solution was added, and 8 μL internal standard stock solution was added; extracted overnight at 4 °C, centrifuged at 12,000 g for 5 min at 4 °C, and the supernatant was taken; the precipitate was re-extracted twice with 5 mL acetonitrile solution, the supernatants were combined, and appropriate amounts of C18 and GCB were added to purify impurities, centrifuged at 12,000 g for 5 min at 4 °C, and the supernatant was taken; dried with nitrogen gas, re-dissolved in 300 μL methanol, filtered through 0.22 μm organic phase membrane, and stored at -20 °C for later detection.

#### 2.3.2 Enzyme activity determination method

##### 2.3.2.1 POD determination

According to the ratio of tissue mass (g): extraction liquid volume (mL) of 1:5-10, approximately 0.1 g tissue was weighed, 1 mL extraction liquid was added, and homogenized in ice bath. Centrifuged at 8,000 g for 10 min at 4 °C, the supernatant was taken and placed on ice for later determination. The microplate reader was preheated for more than 30 min, wavelength adjusted to 470 nm, and zeroed with distilled water. Working solution preparation: reagents were mixed before use; placed at 25 °C for 10 min.

10 μL sample solution and 190 μL working solution were added to a 96-well plate, immediately mixed and timed, recording absorbance A1 at 470 nm at 1 min and A2 at 2 min. Calculate ΔA = A2 - A1.

##### 2.3.2.2 CAT determination

According to the ratio of tissue mass (g): extraction liquid volume (mL) of 1:5-10, approximately 0.1 g tissue was weighed, 1 mL extraction liquid was added, and homogenized in ice bath. Centrifuged at 8,000 g for 10 min at 4 °C, the supernatant was taken and placed on ice for later determination. The microplate reader was preheated for more than 30 min, wavelength adjusted to 405 nm, and zeroed with distilled water. Before determination, 30 μmol/mL standard solution and Reagent 1 were water-bathed at 25 °C for more than 10 min. Mixed well, stood at room temperature for 10 min, and 200 μL was taken to determine absorbance at 405 nm.

##### 2.3.2.3 SOD determination

According to the ratio of tissue mass (g): extraction liquid volume (mL) of 1:5-10, approximately 0.1 g tissue was weighed, 1 mL extraction liquid was added, and homogenized in ice bath. Centrifuged at 8,000 g for 10 min at 4 °C, the supernatant was taken and placed on ice for later determination. The microplate reader was preheated for more than 30 min, wavelength adjusted to 560 nm, and zeroed with distilled water. Reagents were water-bathed for more than 5 min before determination. Mixed well thoroughly, stood at room temperature for 30 min, and absorbance was determined at 560 nm.

#### 2.3.3 TYLCV load detection method

##### 2.3.3.1 Target gene primer sequences

TYLCV-Q-F: TAATCATTTCCACGCCCGTCTC;

TYLCV-Q-R: CAGTATGCTTAATATCATCCCGTTGCTC.

##### 2.3.3.2 DNA extraction

Plant genomic DNA rapid extraction kit was used to weigh and extract DNA from each sample. Finally, 65 μL elution buffer was added for elution, collecting 55 μL DNA. DNA was detected by electrophoresis with 1.5% agarose and 1× TAE electrophoresis buffer. Cloning and sequencing were performed.

##### 2.3.3.3 Real-time fluorescence quantitative PCR detection

DNA samples were diluted 50 times as template for machine detection. Prepared 2× SGExcel FastSYBR Mixture 5 μL, 10 μM primer F 0.2 μL, 10 μM primer R 0.2 μL, RNase-Free ddH2O 3.6 μL, Template (DNA) 1.0 μL. First pre-denatured at 95 °C for 3 min, then 45 cycles (each cycle: denaturation at 95 °C for 15 sec, annealing and extension at 60 °C for 45 sec), and finally melting curve analysis according to instrument guide. The plate with samples was placed in LightCycler 480 II (Roche) for reaction. Relative quantitative analysis was performed according to the method of Livak and Schmittgen (Livak and Schmittgen, 2001).

### 2.4 Statistical analysis

Origin 2024 was used for plotting, and significant differences (P < 0.05) were calculated using SPSS 18 software (SPSS Inc., Chicago, IL, USA).

## 3 Results and analysis

### 3.1 Hormone contents in tomato leaves after different immune elicitor treatments

**Figure 2:**
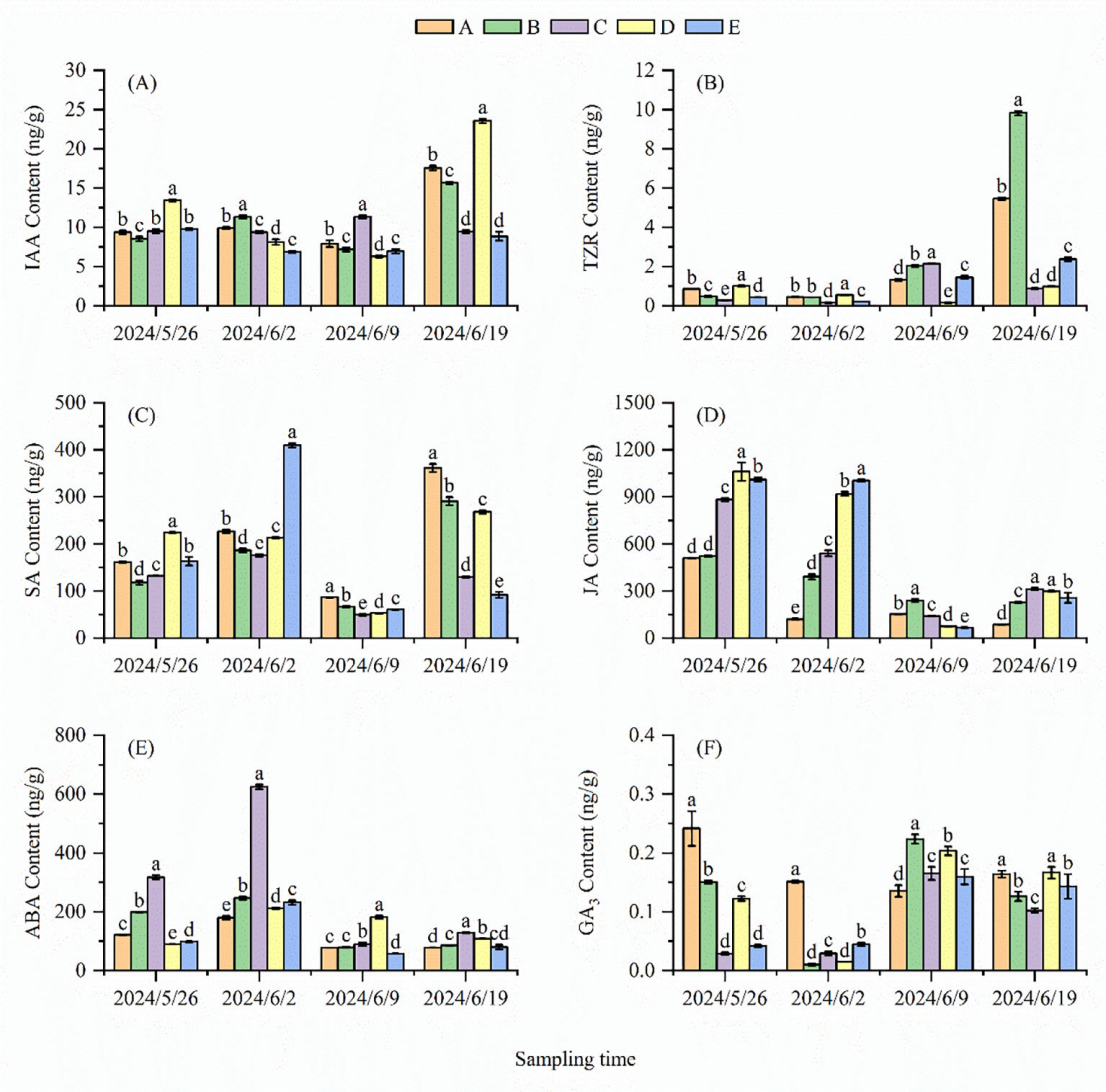
Hormone contents in tomato leaves after different immune elicitor treatments **(A–F)** is IAA, TZR, SA, JA, ABA, and GA_3_ content, respectively. Different lowercase letters after data in the same column indicate significant differences (P < 0.05).

Different immune elicitor treatments had significant effects on the dynamic changes of 6 endogenous hormones (IAA, TZR, SA, JA, ABA, GA_3_) in tomato leaves, with the following response patterns (**Figures 2A–F**):

Indole-3-acetic acid (**Figure 2A**): The clean water control group showed a “slow decrease - slow increase” trend in IAA content, with the 4th sampling close to the initial value. The 5% amino-oligosaccharin (A) and 6% oligosaccharin-chain protein (B) treatment groups showed a “slow increase - slow decrease - significant recovery” trend in IAA content, with the 4th sampling about 5 ng/g higher than the initial value. The 5% S-ABA (C) treatment group showed the same trend as the control. The Kang’erjian Plant Vaccine (D) treatment group showed a “decrease - rapid increase” trend in IAA content, about 10 ng/g higher than the initial value. As a plant growth-promoting hormone, increased IAA content can promote tomato plant vegetative growth, laying a material foundation for disease resistance, indicating that A, B, and D treatments have positive regulatory effects on plant growth.

Trans-zeatin riboside (**Figure 2B**): The A, B treatment groups and control group all showed continuous upward trends in TZR content, with the 4th sampling increasing by 6 ng/g, 10 ng/g, and 2 ng/g respectively compared to initial values. The C and D treatment groups showed no significant changes in TZR content, with final values basically equal to initial values. TZR can promote cell division and robust plant growth. The enhancing effects of A and B treatments indicate that they can strengthen tomato plant vegetative growth potential and improve disease resistance capacity.

Salicylic acid (**Figure 2C**): All treatment groups showed “increase - decrease - re-increase” fluctuation trends in SA content, with A and B treatment groups showing the most significant enhancement effects, increasing by about 200 ng/g at the 4th sampling compared to initial values. The D treatment group increased by about 50 ng/g, while the C group and control group showed no significant enhancement. SA is the core signaling substance of plant systemic acquired resistance, capable of directly activating plant disease defense pathways. The significant induction effects of A and B treatments on SA are important mechanisms for their enhancement of TYLCV resistance.

Jasmonic acid (**Figure 2D**): All treatment groups showed continuous downward trends in JA content, with A and B treatment groups showing the smallest decrease amplitudes of 422.69 ng/g and 295.65 ng/g respectively. The D treatment group and control group showed the largest decrease amplitudes of 763.30 ng/g and 753.77 ng/g respectively. Tomato JA content abnormally increases after TYLCV inoculation, thereby inhibiting plant growth. A and B treatments can significantly reduce the decrease rate of JA, effectively alleviating the growth inhibition effect of virus on plants.

Abscisic acid (**Figure 2E**): All treatment groups showed “first increase - then decrease - tend to flatten” trends in ABA content, with A, B, D treatment groups and control group showing relatively small overall changes. However, the C treatment group showed a rapid increase in ABA content at the 2nd sampling and a sharp decrease at the 3rd sampling, with significant fluctuation amplitude. As a growth-inhibiting hormone, TYLCV infection leads to increased ABA content. Various immune elicitor treatments can reduce ABA accumulation to different degrees, alleviating virus-induced growth inhibition.

Gibberellin (**Figure 2F**): All treatment groups showed “decrease - increase - tend to flatten” trends in GA_3_ content, with A, B, D treatment groups showing higher GA_3_ content at initial stage. By the 4th sampling, there were no significant differences in GA_3_ content among the 5 treatment groups. As an important growth-promoting hormone, there were no significant differences in final GA_3_ content among treatments in this experiment, indicating that different immune elicitors have no significant differential regulatory effects on tomato GA_3_ metabolism.

Comprehensive evaluation of the regulatory effects on 6 endogenous hormones: 5% amino-oligosaccharin (A) and 6% oligosaccharin-chain protein (B) can significantly optimize tomato hormone balance, increase growth-promoting hormones and disease resistance signal hormones, reduce growth-inhibiting hormone levels, and show significantly better therapeutic effects on tomato yellow leaf curl virus disease than other treatments.

### 3.2 Defense enzyme activities in tomato leaves after different immune elicitor treatments

**Figure 3:**
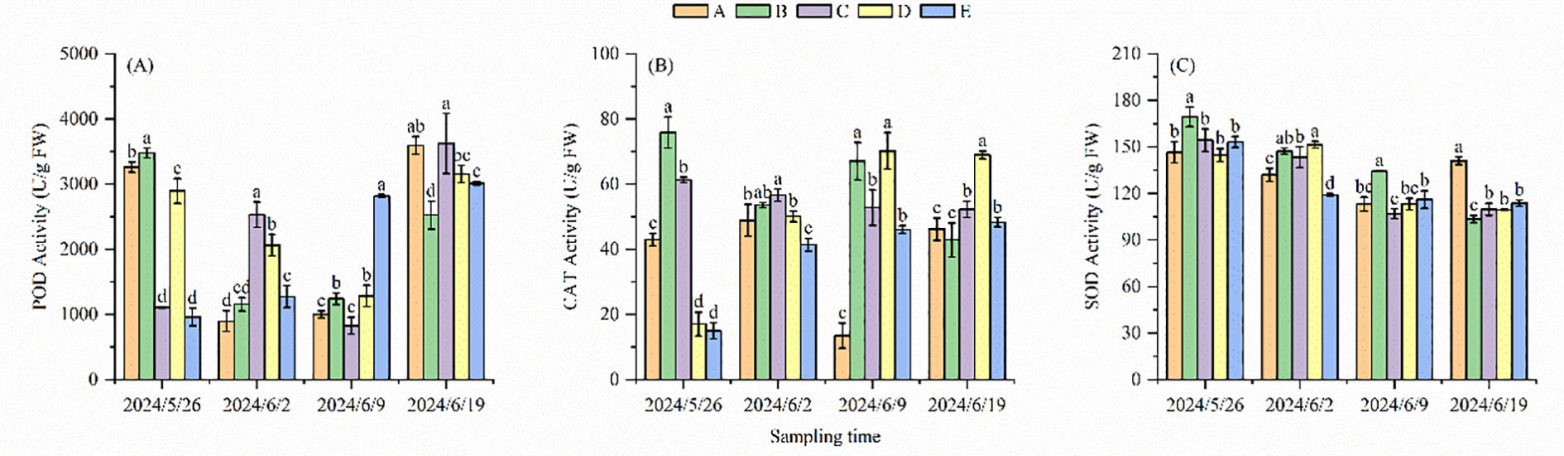
Defense enzyme activities in tomato leaves after different immune elicitor treatments **(A–C)** is POD, CAT, and SOD activity, respectively. Different lowercase letters after data in the same column indicate significant differences (P < 0.05).

Peroxidase (POD), catalase (CAT), and superoxide dismutase (SOD) are core defense enzyme systems in plants for scavenging reactive oxygen species and resisting stress. Their activity changes directly reflect plant stress resistance capacity. The effects of different treatments on 3 enzyme activities are as follows (**Figures 3A–C**):

Peroxidase (**Figure 3A**): At initial sampling, A, B, and D treatment groups showed significantly higher POD activity than C group and control group, subsequently showing “decrease - increase” trends. The control group showed a continuous upward trend in POD activity. By the 4th sampling, POD activities of the 5 treatment groups tended to be similar. As a key enzyme in plant stress response, POD can catalyze reactive oxygen species decomposition. The high initial POD activity of A and B treatments indicates that they can rapidly activate tomato stress defense responses.

Catalase (**Figure 3B**): At initial sampling, B, C, and A treatment groups showed higher CAT activity. CAT activities of all treatment groups gradually became consistent with sampling time progression, with no significant differences at the 4th sampling. CAT synergizes with POD to scavenge hydrogen peroxide. The early enhancement of its activity indicates that A and B treatments can rapidly initiate the plant’s reactive oxygen species scavenging system.

Superoxide dismutase (**Figure 3C**): At initial sampling, there were no significant differences in SOD activity among the 5 treatment groups. With experiment progression, except for the A treatment group where enzyme activity recovered at the 4th sampling, other treatment groups all showed downward trends, with no significant differences among treatment groups throughout. As the first line of defense for reactive oxygen species scavenging, the lack of significant differences in SOD activity in this experiment indicates that different immune elicitors have relatively weak regulatory effects on tomato SOD metabolism.

Comprehensive evaluation of activity changes in 3 defense enzymes: 5% amino-oligosaccharin (A) and 6% oligosaccharin-chain protein (B) can significantly increase activities of key defense enzymes such as POD and CAT at early treatment stages, rapidly activate the plant’s antioxidant defense system, enhance tomato resistance response to TYLCV, and are superior elicitors for regulating tomato defense enzyme systems.

### 3.3 Immune elicitor screening

Combining the regulatory effects of different immune elicitors on tomato leaf endogenous hormone contents and defense enzyme activities, 5% amino-oligosaccharin aqueous solution and 6% oligosaccharin-chain protein wettable powder can significantly optimize tomato hormone metabolism balance, rapidly activate plant defense enzyme systems, effectively enhance tomato resistance to yellow leaf curl virus disease, and are high-efficiency plant immune elicitors screened in this experiment, providing material basis for subsequent control effect experiments.

### 3.4 Control effect experiment on TYLCV

#### 3.4.1 Effects of different treatments on TYLCV disease occurrence

There were significant differences among different chemical treatments in delaying the disease progression of tomato yellow leaf curl virus (TYLCV) and controlling its disease index (**Tables 1**, **2**). The results showed that on September 28 (after two sprays of the agents), only the clean water control group developed infected plants, with a disease index of 0.10, while no disease was observed in any of the other treatments, indicating that all tested agents effectively delayed the initial onset of TYLCV. On October 8 (after three sprays), infected plants appeared in all treatments. The control group had the highest disease index (0.22), while the disease indices of T1, T2, T3, and T4 were 0.10, 0.15, 0.05, and 0.10, respectively. From October 18 to November 8, the number of infected plants continued to increase across all treatments. The control group consistently exhibited the highest disease index, whereas the treated groups showed a significantly slower disease progression rate. Among them, T3 (combination of 5% amino-oligosaccharin + 6% oligosaccharin·chain protein) maintained the lowest disease index throughout. At the end of the experiment (November 8), the disease index of T3 was 0.25, which was lower than that of T1 (5% amino-oligosaccharin alone), T2 (6% oligosaccharin·chain protein alone), and T4 (chemical agent moroxydine-copper acetate). The control efficacy of T3 against TYLCV reached 70.93%.

**Table 1:** Disease index of TYLCV under different treatments.

Treatments	Diseased index				
	Sept. 28	Oct. 8	Oct. 18	Oct. 28	Nov. 8
T1 (5% Amino-oligosaccharin)	0.00	0.10	0.23	0.26	0.35
T2 (6% Oligosaccharin-chain Protein)	0.00	0.15	0.28	0.31	0.39
T3 (Combined)	0.00	0.05	0.12	0.18	0.25
T4 (Moroxydine Hydrochloride-copper, Chemical Control)	0.00	0.10	0.20	0.34	0.38
T5 (Clean Water, Blank Control)	0.10	0.22	0.58	0.81	0.86

**Table 2:** Control effect of TYLCV under different treatments.

Treatments	Control effect				
	Sept. 28	Oct. 8	Oct. 18	Oct. 28	Nov. 8
T1 (5% Amino-oligosaccharin)	100.00	54.55	59.77	67.59	59.30
T2 (6% Oligosaccharin-chain Protein)	100.00	31.82	51.15	61.42	54.94
T3 (Combined)	100.00	77.27	79.89	78.40	70.93
T4 (Moroxydine Hydrochloride-copper, Chemical Control)	100.00	54.55	65.52	58.33	56.40

In conclusion, the combined application of the two plant immune inducers provided significantly better control of TYLCV than either agent alone, while the chemical agent moroxydine-copper acetate showed a control effect comparable to that of the single-inducer treatments.

#### 3.4.2 Effects of different treatments on TYLCV viral load detection

**Figure 4:**
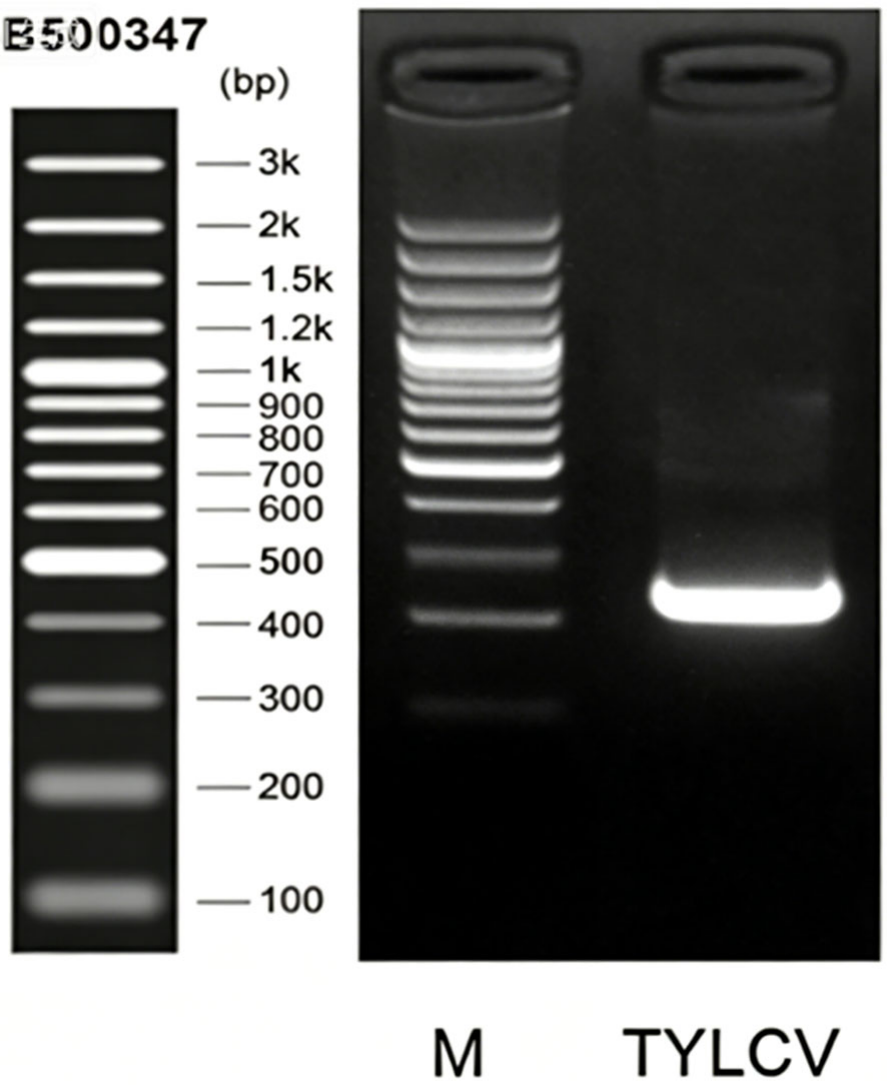
TYLCV conventional PCR detection electrophoresis.

Conventional PCR qualitative detection results (**Figure 4**) showed that TYLCV-positive samples exhibited single, bright specific bands at approximately 3000 bp, consistent with the expected size of TYLCV full genome (2.7-2.8 kb), indicating good PCR amplification specificity and successful detection of TYLCV in all tested samples.

**Figure 5:**
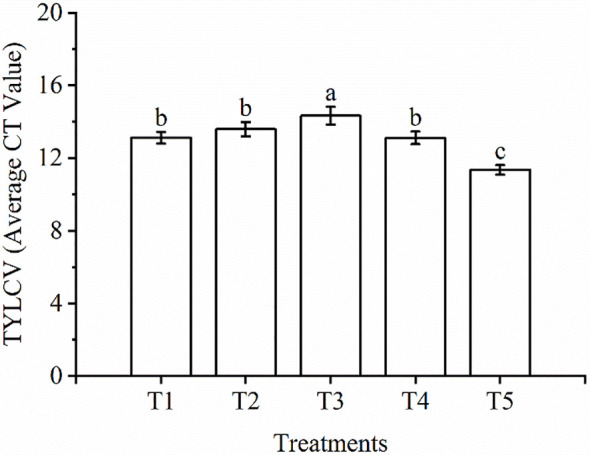
Viral load of tomato yellow leaf curl virus disease under different treatments. Different lowercase letters indicate significant differences (P < 0.05). Viral load is expressed as “mean ± standard deviation”.

Real-time fluorescence quantitative PCR (qPCR) quantitative detection of viral load (**Figure 5**) showed that average CT values of TYLCV in tomato leaves under different treatments had significant differences (P < 0.05). Among them, the combined treatment of 5% amino-oligosaccharin + 6% oligosaccharin-chain protein (T3) had the highest average CT value of 14.34, indicating the lowest viral copy number in plants; the clean water control group had the lowest average CT value of only 11.36, with the highest viral copy number; the 5% amino-oligosaccharin single agent, 6% oligosaccharin-chain protein single agent, and moroxydine hydrochloride-copper treatment groups showed no significant differences in average CT values, all between 13.12-13.60.

According to the conversion relationship between CT values and viral copy numbers, the viral load of the combined treatment group was approximately 7.8 × 10^5 copies/μL, while that of the chemical agent moroxydine hydrochloride-copper treatment group was as high as 4.9 × 10^6 copies/μL, a difference of more than 6 times. The results fully demonstrate that combined use of 5% amino-oligosaccharin and 6% oligosaccharin-chain protein can significantly inhibit TYLCV proliferation in tomato plants and is the optimal agent combination for TYLCV control.

## 4 Discussion

### 4.1 Physiological mechanisms of plant immune elicitors regulating tomato resistance to TYLCV

Plant resistance to viral diseases results from the coordinated action of various physiological processes including endogenous hormone metabolism and defense enzyme system activation. The present study specifically targeted monopartite TYLCV, which is the predominant form causing epidemics in Chinese tomato production systems (Czosnek and Laterrot, 1997; Moriones and Navas-Castillo, 2000). This study found that 5% amino-oligosaccharin and 6% oligosaccharin-chain protein can enhance tomato resistance to TYLCV through dual pathways of hormone regulation and defense enzyme activation, consistent with previous research results on immune elicitors controlling plant viral diseases (Su et al., 2004; Sun et al., 2014; Wang et al., 2020).

Hormonal signaling integration and redox crosstalk. The observed changes in SA, JA, and ABA suggest complex regulatory interactions that are central to plant immune responses. Recent studies have highlighted the intricate crosstalk between these hormones and reactive oxygen species (ROS) in orchestrating plant defense networks (Ali et al., 2025b; Parveen et al., 2022). Salicylic acid, as the core signaling molecule of plant systemic acquired resistance, can activate expression of downstream disease resistance-related genes and induce broad-spectrum disease resistance in plants (Li et al., 2010; Yan et al., 2018). In this experiment, 5% amino-oligosaccharin and 6% oligosaccharin-chain protein treatments could significantly increase SA content in tomato leaves, indicating that they can enhance tomato systemic acquired resistance to TYLCV by activating SA-mediated disease resistance signaling pathways. This aligns with research demonstrating that SA-mediated defense responses are modulated by cellular redox status, with low ascorbate abundance triggering changes in hormone sensitivity that steer adaptive plant responses toward SA defenses (Foyer et al., 2012).

The integration of hormone and redox signaling is further evidenced by the JA and ABA dynamics observed in this study. While SA typically antagonizes JA-dependent defenses to prioritize SA-dependent resistance, this regulatory framework is profoundly influenced by ROS-mediated signaling networks (Ali et al., 2025b; Parveen et al., 2022). The ability of amino-oligosaccharin and oligosaccharin-chain protein treatments to significantly reduce JA decrease rates while maintaining ABA homeostasis suggests that these elicitors activate a coordinated hormonal-redox response that optimizes the balance between growth and defense.

Auxin-driven resilience pathways. Notably, this study demonstrated that both elicitors significantly increased IAA content, providing evidence for the involvement of auxin-driven resilience pathways in TYLCV resistance. Recent comprehensive reviews have established that auxins play multifaceted roles in enabling plants to cope with environmental stresses by regulating growth and activating defense mechanisms. The TIR1/AFB-Aux/IAA-ARF signaling cascade, central to auxin perception, has been shown to interact extensively with stress-responsive pathways, including those mediated by SA, JA, and ABA. Specifically, auxin signaling can enhance resistance against viral infections by inducing the transcription of specific defense-related genes (Ali et al., 2025b).

The crosstalk between auxin and ROS is particularly relevant to the observed enhancement of POD and CAT activities in this study. Research has demonstrated that auxin homeostasis in specific tissues is crucial for regulating H_2_O_2_ production through altered expression of antioxidant enzymes. The auxin signaling mutant tir1afb2 exhibits reduced accumulation of H_2_O_2_ and superoxide anion, alongside enhanced activities of catalase and ascorbate peroxidase, indicating that optimal auxin signaling is required for precise ROS modulation during stress responses (Tognetti et al., 2011). The elevated IAA levels observed in amino-oligosaccharin and oligosaccharin-chain protein treated plants likely contribute to the enhanced POD and CAT activities through auxin-ROS crosstalk, thereby establishing a robust antioxidant defense system against TYLCV-induced oxidative stress.

Antioxidant enzyme activation and ROS homeostasis. In terms of defense enzyme systems, peroxidase and catalase are key enzymes for scavenging reactive oxygen species in plants. Viral infection leads to massive accumulation of reactive oxygen species in plants, causing cellular oxidative damage, while defense enzyme system activation can effectively scavenge reactive oxygen species and alleviate oxidative stress (Zhang et al., 2024). In this experiment, 5% amino-oligosaccharin and 6% oligosaccharin-chain protein could significantly increase POD and CAT activities at early treatment stages, rapidly initiate plant antioxidant defense responses, and reduce cellular damage caused by TYLCV infection.

The rapid activation of POD and CAT observed in this study aligns with recent advances in understanding ROS-mediated defense networks. ROS, including superoxide anion, hydrogen peroxide, and hydroxyl radicals, are generated through various enzymatic pathways and serve dual functions: inhibiting pathogen growth while activating defense-related gene expression as signaling molecules. The oxidative burst triggered by pathogen recognition initiates hypersensitive response and facilitates the establishment of systemic acquired resistance by inducing systemic signaling networks. The interplay between ROS and phytohormones such as JA, SA, and auxin further complicates this regulatory framework, underscoring the importance of coordinated hormonal-redox signaling in orchestrating both local and systemic defense responses (Ali et al., 2025a). The lack of significant differences in superoxide dismutase (SOD) activity among treatments suggests that SOD may not be a core enzyme in tomato TYLCV defense response, or that elicitor regulatory effects on it have time- or concentration-dependent characteristics requiring further experimental verification.

Comprehensive hormonal balance optimization. Meanwhile, the two elicitors could significantly increase IAA and TZR contents, reduce levels of growth-inhibiting hormones such as jasmonic acid and abscisic acid, optimize tomato endogenous hormone balance, and promote plant vegetative growth. As an important growth-promoting hormone, GA_3_ showed no significant differences in final content among treatments, indicating it is not a key hormone for the two elicitors to regulate (Ali et al., 2025a) tomato TYLCV resistance, providing direction for subsequent in-depth research on elicitor action targets.

### 4.2 Synergistic control effects of amino-oligosaccharin and oligosaccharin-chain protein combination

This study found that combined use of 5% amino-oligosaccharin and 6% oligosaccharin-chain protein showed significantly better control effects on tomato TYLCV than single treatments, manifested as the fewest diseased plants and lowest viral load, showing obvious synergistic enhancement effects.

Dual-receptor recognition and signal amplification. Amino-oligosaccharin, a deacetylation product of chitin, can be recognized by pattern recognition receptors (PRRs) on the plant cell membrane. Studies have shown that in tomato, *Bti9* (also known as *SlLYK13*), a homolog of chitin elicitor receptor kinase 1 (*CERK1*), is a key component of chitin signaling, responsible for recognizing chitin oligosaccharides and triggering downstream immune responses. Amino-oligosaccharin activates plant pattern-triggered immunity (PTI) by binding to CERK1, leading to a burst of reactive oxygen species (ROS), calcium influx, and activation of the mitogen-activated protein kinase (MAPK) cascade (Sun et al., 2025). Oligosaccharin·chain protein consists of oligosaccharide fragments and a disease resistance protein; the resistance protein component may be recognized by plants through a receptor system different from that for amino-oligosaccharin. Proteinaceous elicitors typically initiate calcium signaling and ROS production via receptor kinases or direct action on the lipid bilayer of the cell membrane (Popa et al., 2026). Thus, the two elicitors may simultaneously activate plant immune perception through distinct receptor systems, producing a “dual-receptor recognition” effect that amplifies the initial input of immune signals.

Enhanced MAPK signaling and transcriptional regulation. Studies indicate that the synergistic action of different elicitors can alter MAPK phosphorylation dynamics and enhance immune responses. The combination of amino-oligosaccharin and oligosaccharin-chain protein may simultaneously activate multiple MAPK cascades, thereby achieving a superposition of signaling pathways. Specifically, amino-oligosaccharin primarily signals through the CERK1-RLCK-MAPK3/6 pathway, whereas oligosaccharin-chain protein may enhance signal transduction through additional MAPK modules. Their synergy can form a stronger immune response network (Greco et al., 2025).

The integration of these signaling pathways likely converges on WRKY transcription factors, which serve as central nodes in the hormonal-redox signaling network. WRKY TFs mediate PTI cascades downstream of MAPK signaling and are modulators of plant tolerance to biotic stress and systemic acquired resistance (Zhang et al., 2025). These TFs adjust ET, JA, and SA interactive pathways, and control developmental processes through cytokinin and auxin, thereby coordinating the complex defense response required for TYLCV resistance.

Strengthened SA signaling through parallel pathways. Salicylic acid (SA) is a core signaling molecule in plant systemic acquired resistance (SAR). In this study, single and combined treatments of amino-oligosaccharin and oligosaccharin·chain protein all significantly increased SA content in tomato leaves. Research has shown that the antiviral resistance induced by amino-oligosaccharin primarily depends on the SA-mediated signaling pathway, characterized by upregulation of the pathogenesis-related protein 1 (PR1) gene (Jia et al., 2016). Oligosaccharin·chain protein may enhance SA signaling through additional mechanisms. Proteinaceous elicitors can induce activation of calcium-dependent protein kinases (CDPKs), which then phosphorylate and activate WRKY transcription factors, forming an SA signal amplification loop parallel to NPR1 (Sun et al., 2025). Therefore, the combined treatment may strengthen SA signal transduction through both NPR1-dependent and WRKY-dependent mechanisms, achieving a “signal pathway superposition” effect.

The superior performance of the combined elicitor treatment over chemical agents can be attributed to its ability to activate multiple layers of plant immunity while maintaining hormonal and redox homeostasis. The chemical agent moroxydine-copper acetate, while providing some antiviral effect, does not induce the comprehensive systemic resistance and sustained antioxidant defense observed with the elicitor combination. This highlights the advantage of plant immune inducers in engaging natural defense networks that provide broad-spectrum, long-lasting protection against viral diseases.

### 4.3 Application prospects of plant immune elicitors in green control of TYLCV

The green development of the tomato industry requires reducing the use of chemical pesticides and promoting environmentally friendly disease control technologies. Plant immune inducers, owing to their advantages of no residues, low risk of resistance development, and ability to improve crop quality, have become ideal agents for the green control of tomato TYLCV (Wang et al., 2025b). Both 5% amino-oligosaccharin and 6% oligosaccharin·chain protein screened in this study are bio-sourced inducers. Their combined application can significantly reduce the incidence of TYLCV in autumn tomato cultivation, and the method is simple (foliar spray, three consecutive applications), making it suitable for promotion and adoption by farmers.

Currently, the application of plant immune inducers still faces some challenges, such as their efficacy being greatly influenced by crop variety, growth stage, environmental conditions, etc., and the induced resistance having a certain duration (Yu et al., 2013). In the future, this inducer combination can be integrated with biological control measures (e.g., releasing the whitefly natural enemy Encarsia formosa) to build a more efficient and environmentally friendly integrated control system for tomato TYLCV, promoting high-quality and sustainable development of the tomato industry.

### 4.4 Limitations of this study and future research directions

This study only used the tomato variety “Nongbo Pink 18109” as experimental material, and the applicability of its results to other tomato varieties still requires verification. Based on the current findings, it is plausible that the elicitor combination may exhibit differential effects depending on the variety’s baseline susceptibility to TYLCV. For highly susceptible varieties, the induced resistance might be more noticeable due to a stronger contrast with untreated controls, whereas moderately resistant or tolerant varieties may show a more limited incremental benefit, as they already possess partial defense mechanisms. Conversely, varieties with inherent resistance could potentially respond more robustly to immune inducers if their genetic background facilitates signal amplification. Therefore, further studies across a range of tomato genotypes with varying TYLCV susceptibility levels are needed to delineate the variety-specific efficacy of this elicitor combination.

Meanwhile, experiments were only conducted under greenhouse conditions with insect-proof nets. Although nets were installed, whitefly penetration still occurred, contributing to virus spread. In open-field environments, whitefly pressure is typically much higher due to unrestricted pest movement, larger population sizes, and less controlled microclimates. Under such conditions, the efficacy of foliar-applied immune inducers alone may be compromised, as continuous virus inoculation from immigrating whiteflies can overwhelm induced resistance. Consequently, the application strategy should be adjusted. Integration with systemic insecticides is likely necessary to reduce vector population and virus transmission at the source. Additionally, more frequent or higher-concentration applications of the elicitor combination might be required to maintain elevated defense levels. A practical integrated approach could combine the elicitor spray schedule with timely insecticide applications, reinforced physical barriers (e.g., high-density nets or insect-proof screens), and cultural practices such as reflective mulches or trap crops. Further field trials are essential to optimize this integrated strategy under real-world, high-whitefly-pressure conditions.

Future research can be expanded from the following aspects: ① Conduct elicitor control effect experiments across different tomato varieties and cultivation modes to refine technical parameters; ② Explore the combined application of elicitors with biological pesticides, physical insect prevention measures, and systemic insecticides to develop comprehensive integrated pest management (IPM) protocols;③ Analyze the molecular mechanisms by which the combination of amino-oligosaccharin and oligosaccharin-chain protein regulates tomato resistance to TYLCV, with particular focus on the integration of hormone signaling, ROS homeostasis, and defense gene activation, providing a theoretical basis for the efficient utilization of immune inducers.

## Data Availability

The raw data supporting the conclusions of this article will be made available by the authors, without undue reservation.

## References

[B1] Ali M. Kaderbek T. Khan M. A. Skalicky M. Brestic M. Elsabagh M. . (2025a). Biosynthesis and multifaceted roles of reactive species in plant defense mechanisms during environmental cues. Plant Stress 18. doi: 10.1016/j.stress.2025.101102. PMID: 38826717

[B2] Ali M. Shi L. Khan M. A. Ali A. Hu S. Shen J. (2025b). Auxin biodynamics and its integral role in enhancing plant resilience to environmental cues. Physiol. Plant 177, e70165. doi: 10.1111/ppl.70165. PMID: 40114288

[B3] Bai X. Li Y. Wang G. Yin Q. Geng B. Dong W. Molecular Identification and Sequence Analysis of Tomato yellow leaf curl virus (TYLCV) Isolate from Shijiazhuang. (2015). Acta Horticulturae Sinica, 42(1), 167.

[B4] Czosnek H. Laterrot H. (1997). A worldwide survey of tomato yellow leaf curl viruses. Arch. Virol. 142, 1391–1406. doi: 10.1007/s007050050168. PMID: 9267451

[B5] Foyer C. H. Kerchev P. I. Hancock R. D. (2012). The ABA-INSENSITIVE-4 (ABI4) transcription factor links redox, hormone and sugar signaling pathways. Plant Signaling Behav. 7, 276–281. doi: 10.4161/psb.18770. PMID: 22415048 PMC3404864

[B6] Greco M. Coculo D. Conti A. Agresti S. Pontiggia D. Mélida H. . (2025). Biorefining of anaerobic digestates for the recovery of biostimulants and bioelicitors for immune priming and plant protection. Environ. Sci. Technol. 59, 21700–21714. doi: 10.1021/acs.est.5c03321. PMID: 41026933 PMC12529957

[B7] Huang L. Tang Y. Wang S. Chen J. Du J. Yan S. . (2025). Dufulin impacts plant defense against tomato yellow leaf curl virus infecting tomato. Viruses 17, 53. doi: 10.3390/v17010053. PMID: 39861842 PMC11768724

[B8] Huang X. Wei J. Wu D. Mi N. Fang S. Xiao Y. . (2022). Silencing of SlDRB1 gene reduces resistance to tomato yellow leaf curl virus (TYLCV) in tomato (Solanum lycopersicum). Plant Signaling Behav. 17, 1. doi: 10.1080/15592324.2022.2149942. PMID: 36453197 PMC9718546

[B9] Ji Z. Wang X. Wang R. Dong J. Xu J. Tong Y. . (2017). Effect of paclobutrazol on control of peach shoot blight. J. Yangzhou University(Agricultural Life. Sci. Edition) 38, 99–104. doi: 10.16872/j.cnki.1671-4652.2017.04.018

[B10] Jia X. Meng Q. Zeng H. Wang W. Yin H. (2016). Chitosan oligosaccharide induces resistance to Tobacco mosaic virus in Arabidopsis via the salicylic acid-mediated signalling pathway. Sci. Rep. 6. doi: 10.1038/srep26144. PMID: 27189192 PMC4870575

[B11] Kheyr-Pour A. Bendahmane M. Matzeit V. Accotto G. P. Gronenborn B. (1991). Tomato yellow leaf curl virus from Sardinia is a whitefly-transmitted monopartite geminivirus. Nucleic Acids Res. 19, 6763–6769. doi: 10.1093/nar/19.24.6763. PMID: 1840676 PMC329307

[B12] Lefeuvre P. Martin D. P. Harkins G. Lemey P. Heydarnejad J. (2010). The spread of tomato yellow leaf curl virus from the Middle East to the world. PloS Pathog. 6, e1001164. doi: 10.1371/journal.ppat.1001164. PMID: 21060815 PMC2965765

[B13] Li W. Sun G. Yang W. Lin N. Li K. Liu F. . (2010). Living with temperature changes: Salicylic acid at the crossroads of plant immunity and temperature resilience. Sci. Adv. 11, 12. doi: 10.1126/sciadv.ady3327. PMID: 40929268 PMC12422185

[B14] Liu X. Liang X. Cai Z. Liu Z. Wang X. Zhou C. . (2026). BioCNTs mediated delivery of systemically mobile small RNAs via leaf spray to control both tomato DNA and RNA viruses. Adv. Sci. 13, e04889. doi: 10.1002/advs.202504889. PMID: 41422436 PMC12915072

[B15] Liu Y. Ma J. Li J. Wang X. Liang X. Ji Y. . (2023). Integration and innovation of key technologies for monitoring, early warning, and green prevention and control of the tomato leaf miner (Tuta absoluta) in Ningxia and their application.

[B16] Livak K. J. Schmittgen T. D. (2001). Analysis of relative gene expression data using real-time quantitative PCR and the 2(-Delta Delta C(T)) method. Methods (San Diego Calif.) 25, 402–408. doi: 10.1006/meth.2001.1262. PMID: 11846609

[B17] Moriones E. Navas-Castillo J. (2000). Tomato yellow leaf curl virus, an emerging virus complex causing epidemics worldwide. Virus Res. 71, 123–134. doi: 10.1016/s0168-1702(00)00193-3. PMID: 11137167

[B18] Nicaise V. (2014). Crop immunity against viruses: outcomes and future challenges. Front. Plant Sci. 5, 18. doi: 10.3389/fpls.2014.00660. PMID: 25484888 PMC4240047

[B19] Parveen N. Kandhol N. Sharma S. Singh V. P. Chauhan D. K. Ludwig-Müller J. . (2022). Auxin crosstalk with reactive oxygen and nitrogen species in plant development and abiotic stress. Plant Cell Physiol. 63, 1814–1825. doi: 10.1093/pcp/pcac138. PMID: 36208156

[B20] Popa A. Zugravu M.-M. Israel-Roming F. (2026). Application of plant defence elicitors in fruit crop protection with a one health approach. Agnoromy. 16(5), 590. doi: 10.3390/agronomy16050590

[B21] Su X. Wang Y. Jia L. Liu W. (2004). Control effect of chitosan-oligosacchrides on main diseases of tomato in Shaanxi. Acta Agric. Boreali-Occident Sin. 13(2), 79–82. doi: 10.7606/j.issn.1004-389.2004.2.020

[B22] Sun G. Peng C. Liu Y. Deng J. Yuan H. (2014). Study on control effect of amino oligosaccharins against tomato late blight. Pesticide Sci. Administration 35, 60–62. doi: 10.3969/j.issn.1002-5480.2014.12.014

[B23] Sun W. Lv W. Li L. Yin G. Hang X. Xue Y. . (2016). Eugenol confers resistance to tomato yellow leaf curl virus (TYLCV) by regulating the expression of SlPer1 in tomato plants. N. Bio/Technol. 33, 345–354. doi: 10.1016/j.nbt.2016.01.001. PMID: 26776605

[B24] Sun G. Xiao Y. Yin H. Yu K. Wang Y. Wang Y. (2025). Oligosaccharide elicitors in plant immunity: Molecular mechanisms and disease resistance strategies. Plant Commun. 6. doi: 10.1016/j.xplc.2025.101469. PMID: 40754821 PMC12744754

[B25] Tognetti V. B. Mühlenbock P. Van Breusegem F. (2011). Stress homeostasis - the redox and auxin perspective. Plant Cell Environ. 35, 321–333. doi: 10.1111/j.1365-3040.2011.02324.x. PMID: 21443606

[B26] Walters D. R. Ratsep J. Havis N. D. (2013). Controlling crop diseases using induced resistance: challenges for the future. J. Exp. Bot. 64, 1263–1280. doi: 10.1093/jxb/ert026. PMID: 23386685

[B27] Wang D. Fan X. Dong L. Li Y. Xue Y. Li H. . (2026). Brassinolide application mitigates blossom-end rot in tomato by enhancing calcium homeostasis and antioxidant defense under calcium deficiency. Plants-Basel 15, 427. doi: 10.3390/plants15030427. PMID: 41681591 PMC12899996

[B28] Wang Z. Li X. Chang X. Cai X. Yang X. Chen J. . (2025b). Analysis of expression level of resistance related genes induced by elicitors and their control efficacy against rice false smut. Jiangsu Agric. Sci. 53, 123–129. doi: 10.15889/j.issn.1002-1302.2025.18.017

[B29] Wang D. Li Y. Zhang Q. Li S. Pang Y. Ma K. . (2023). Effects of different microbial treatments on tomato soil microbial diversity. Xinjiang Agric. Sci. 60, 2248–2257. doi: 10.5846/stxb201902150270

[B30] Wang L. Sun Y. Zhao J. Wang W. Xu B. Wang G. (2016). Effect of different concentrations of PP333 on growth of pepper seedlings. J. Anhui Agric. Sci. 44, 14–15. doi: 10.13989/j.cnki.0517-6611.2016.34.005

[B31] Wang S. Wang F. Ma J. Liu Y. Zhu L. Xu X. (2020). Control effects of five pesticides on pepper virus disease. J. Zhejiang Agric. Sci. 61, 463–464. doi: 10.16178/j.issn.0528-9017.20200323

[B32] Wang D. Zhang C. Chen C. Wang Z. Guo J. Liu X. . (2025a). Physiological and molecular mechanisms of 2,4-D in enhancing tomato resistance to tomato yellow leaf curl virus disease. Jiangsu Agric. Sci. 53, 130–136. doi: 10.15889/j.issn.1002-1302.2025.18.018

[B33] Yan Y. Pan C. Du Y. Li D. Liu W. (2018). Exogenous salicylic acid regulates reactive oxygen species metabolism and ascorbate-glutathione cycle in Nitraria tangutorum Bobr. under salinity stress. Physiol. Mol. Biol. Plants 24, 577–589. doi: 10.1007/s12298-018-0540-5. PMID: 30042614 PMC6041230

[B34] Yin W. Chen H. Huang Y. Ha S. Bie Z. (2016). Effects of different concentration of PP_(333) on the quality of watermelon seedlings. China Cucurbits Vegetables 29, 41–45. doi: 10.16861/j.cnki.zggc.2016.0213

[B35] Yu L. Sun J. Guo S. Yan J. Zhu W. (2013). Resistance to tomato yellow leaf curl virus in tomato induced by benzothiadiazole. Jiangsu J. Agric. Sci. 29, 71–75. doi: 10.3969/j.issn.1000-4440.2013.01.012

[B36] Zhang X. Tong J. Bai A. Liu C. Xiao L. Xue H. (2020). Phytohormone dynamics in developing endosperm influence rice grain shape and quality. J. Integr. Plant Biol. 62, 175–187. doi: 10.1111/jipb.12927. PMID: 32198820

[B37] Zhang C. Wang D. Li Y. Wang Z. Wu Z. Zhang Q. . (2024). Gibberellin positively regulates tomato resistance to tomato yellow leaf curl virus (TYLCV). Plants-Basel 13, 1277. doi: 10.3390/plants13091277. PMID: 38732492 PMC11085062

[B38] Zhang L. Wang Y. Ni Z. Yu Y. (2025). Functional and mechanistic insights into plant VQ proteins in abiotic and biotic stress responses. Plants 14, 3855. doi: 10.3390/plants14243855. PMID: 41470737 PMC12736750

